# Manager characteristics and the informativeness of banks’ loan loss provisioning

**DOI:** 10.1007/s11142-025-09905-4

**Published:** 2025-07-17

**Authors:** Jannis Bischof, Nicolas Rudolf

**Affiliations:** 1 Mannheim Business School, University of Mannheim, Mannheim, Germany; 2 HEC Lausanne, University of Lausanne, Lausanne, Switzerland

**Keywords:** M14, M41, M48, M52, G20, Manager Characteristics, Bank Governance, Top Management Team, Loan Loss Provisions, Timeliness, Upper Echelons Theory, AKM Method

## Abstract

This study investigates the role of individual managers in banks’ financial reporting. We exploit the connectedness between different managers and find that individual bank managers explain approximately 19 percent of banks’ loan loss provisions. This observation is consistent with the substantial reporting discretion that individual bank managers use in the estimation of loan loss provisions and that is increasingly subject to financial stability concerns by prudential supervisors. Our results suggest that these concerns are valid, as individual management discretion is associated with greater discretionary loan loss provisions and proxies for opportunistic accounting, especially the reduction in the timeliness of these provisions and the lesser degree to which the allowance for credit losses maps into future charge-offs. These findings are relevant for the design of regulatory measures aimed at limiting the managerial influence on accounting choices in banking and can inform debates on the desirability of discretion within the reporting process of banks.

## Introduction

Does individual manager discretion shape the informativeness of banks’ loan loss provisions? Individual managers significantly influence corporate actions, such as risk-taking and capital structure choices (Bertrand and Schoar 2003; Graham et al. 2013). Their incentives and preferences are associated with the corporate reporting choices of nonfinancial firms and significantly influence their reporting quality (Bamber et al. 2010; Davis et al. 2015; Ge et al. 2011; Wells 2020). However, scant evidence exists on whether individual managers have a similar impact on the financial reporting of banks, for which loan loss provisions represent the most important accrual (Beatty and Liao 2014). Because of the subjectivity of the management estimates in loan loss provisioning and thus the unique attributes of banks’ accruals (Stubben 2010), it is unclear whether the economic magnitude of the prior results from nonfinancial industries extends to the banking setting and thus whether managers’ discretion influences the informativeness of the loan loss provisions.

The informativeness of loan loss provisions has significant implications for bank stability (Acharya and Ryan 2016) and has been at the core of the regulatory debate since the global financial crisis of 2008–2009 (e.g., Acharya and Ryan 2016; Bischof et al. 2021, 2025; Bushman and Williams 2012; Kim 2022; Kothari and Lester 2012; Wheeler 2019). This debate has also motivated recent regulations concerning manager appointments in banking.^1^ Against this background, it is important to examine individual managers’ influence on critical bank policies, such as the loan loss provisioning choice. While the influence of individual managers on bank actions and accounting choices originates from their personal characteristics and preferences, a bank’s governance also shapes their decision-making (e.g., Aggarwal et al. 2009; Anginer et al. 2018; Cornett et al. 2008; Ellul and Yerramilli 2013), including the interactions with other managers of the top management team (Garlappi et al. 2017; Hambrick et al. 1996; Hambrick 2007). Evidence from other industries generally suggests that governance mechanisms mute discretionary financial reporting choices (e.g., Karamanou and Vafeas 2005; Li and Wahid 2018; Qi et al. 2018; Zhang 2019). Given the unique regulatory environment and risk-shifting incentives in banking (Bushman 2014; Laeven 2013), it is less clear whether governance can constrain individual manager discretion in banks’ loan loss provisioning decisions to the same extent.

To address these questions, we build on research in the fields of accounting (Bushman et al. 2018; Wells 2020) and finance (Malmendier et al. 2011; Nguyen et al. 2017; Schoar et al. 2024) that investigates the influence of manager characteristics on corporate policies. We construct a comprehensive dataset of the top executives of US banks between 1993 and 2021. The dataset contains information about manager characteristics (e.g., compensation, education, and experience), bank characteristics (e.g., size, risk, and performance), and loan loss provisioning choices. In the first step of our analysis, we test for an association between loan loss provisions and idiosyncratic manager characteristics. We capture managers’ total influence on loan loss provisions through a fixed-effects structure that exploits the interconnectedness between managers who switch to other sample banks and managers who remain at the same bank (Abowd et al. 1999; hereafter AKM method). These fixed effects are thought to capture latent time-invariant management styles that reflect preferences for observable management choices, even if the underlying factors explaining those choices remain unobservable. We then analyze how the role of idiosyncratic management styles in the choice of loan loss provisions relates to other bank actions. To this end, we compare the time-invariant management styles that manifest in the choice of loan loss provisions with other management choices that affect a bank’s risk-taking (such as leverage or loan quality).

In the second step of the analysis, we explore whether it matters for the informativeness of banks’ loan loss provisions when individual managers strongly influence this accounting choice. Therefore, we first test whether a manager’s provisioning style relates systematically to the discretionary part of a bank’s loss provisions. Because managers can exercise this discretion to convey private information about loan quality or for opportunistic reasons, we next examine whether discretion in loan loss provisioning is associated with the informativeness of provisions. Specifically, we employ two models from prior literature (Beatty and Liao 2011; Bushman and Williams 2015) to test whether managerial discretion is associated with the timeliness of provisions. To supplement these analyses, we examine the association between the discretion of individual managers and the mapping of the allowance for credit losses into future loan charge-offs (e.g., Beck and Narayanamoorthy 2013).

In the third and final step, we exploit cross-sectional variation in the corporate governance of banks to explore whether the impact of potentially opportunistic management discretion on the informativeness of loan loss provisions is muted when governance arrangements facilitate monitoring. We employ four proxies for effective governance, such as a bank’s board composition and independence, serving as indicators of tighter checks and balances within the bank (Bebchuk and Cohen 2005; Coles et al. 2014; Cremers et al. 2017; Evans et al. 2025; Gompers et al. 2003).

Our results show that managers exert their influence in an idiosyncratic way through their preferences, skills, or talent, which are notoriously hard to measure but key to understanding their role in the accounting process. Consistent with this notion, we find that the time-invariant, unobservable attributes of individual managers account for 18.8 percent of this variation, that is, more than twice as much as unobservable bank attributes (8.2 percent). This seems plausible when we compare our results to prior literature documenting that individual managers explain about 27.5% of the variation of nonperforming loans (Hagendorff et al. 2021—25.0% in our sample).

The influence of manager discretion is consistent with the complexity and subjectivity inherent in loan loss provisioning (e.g., Beatty and Liao 2014).^2^ The identified management styles are economically meaningful, and the direction of underlying preferences for certain bank policies and loan loss provisions appears systematic. For example, managers with more discretion over loan loss provisioning decisions also exhibit a preference for greater risk-taking, that is, managers who have a distinct impact on loan loss provisioning also influence other bank actions, especially those for risk-taking, lending, and compensation, in a systematically related way. Put differently, a manager has a personal provisioning style that is bundled with these other management decisions.

While these results are consistent with the idiosyncratic influence that managers have on loan loss provisions, it is still an open question whether their influence is opportunistic or informative. To this end, we compare the properties of loan loss provisions in banks that have high-influence managers with those that have low-influence managers. We first document that managers with a high influence over loan loss provisions are associated with greater discretionary accruals than low-influence managers. Further results suggest that managers with high influence over loan loss provisions are also associated with less timely provisions and lower predictability of future charge-offs. Managers with a higher influence over loan loss provisions thus tend to exercise their discretion more opportunistically and less so to reveal their proprietary information.

To add some context, the average loan loss provision in our sample corresponds to 0.7% of banks’ total assets. The average fraction of discretionary provisions lies in the range between 31.1% and 73.8% of total provisions, that is, between 0.21% and 0.52% of total assets (depending on the model). Our results suggest that a manager with an above-average influence on loan loss provisions is associated with an increase in discretionary loan loss provisions by 0.4 percentage points; that is, the discretionary provisions increase from 0.21% to 0.61% (at the lower bound of the estimate for discretionary accruals) or from 0.52% to 0.92% of total assets (at the upper bound). The magnitude of the difference between a low- and a high-influence manager thus appears economically meaningful.

However, we also find evidence that specific governance mechanisms attenuate the significant association between opportunistic provisioning styles and the informativeness of loan loss provisioning. Effective governance, specifically board independence, low insider ownership, and top management team gender diversity, appear to reduce individual managers’ influence over the informativeness of loan loss provisions, suggesting that institutional features can serve as a mitigating factor against opportunistic provisioning preferences of particular managers.

Our study contributes to the literature on the role of individual managers in banks’ corporate reporting. In particular, we show how much variation in banks’ loan loss provisions is explained by individual managers and how this relates to the timeliness and informativeness of loan loss provisions. The literature on banks’ loan loss provisioning choices goes back to at least Beaver et al. (1989) and has documented various bank-specific incentives, such as capital market pressure, private ownership, taxation, and regulation (e.g., Ahmed et al. 1999; Beatty et al. 1995, 2002; Bushman and Williams 2012; Collins et al. 1995; Kanagaretnam et al. 2004) as well as variation over time (e.g., Beck and Narayanmoorthy 2013; Liu and Ryan 2006; Lopez-Espinosa et al. 2021). However, we know little about the impact of individual bank managers on provisioning choices (Beatty and Liao 2014). Not least because of the particularly subjective nature of the loan loss accruals, it is unclear whether the more specific evidence concerning the influence of managers on accounting policies in nonfinancial firms (e.g., Bamber et al. 2010; Bertrand and Schoar 2003; Wells 2020) extends to loan loss provisioning choices by bank managers.

While prior literature has documented the influence of individual bank managers over other bank policies, such as risk management (Cronqvist et al. 2012; Hagendorff et al. 2021; Schoar and Zuo 2017) or loan contract design (Bushman et al. 2021; Cerqueiro et al. 2011; Costello et al. 2020; Herpfer 2021), studies on loan loss provisioning tend to neglect this influence, approximate manager idiosyncrasies with indirect proxies such as bank size (e.g., Beatty and Liao 2011; Acharya and Ryan 2016), or focus on very specific manager characteristics, such as crisis experience (Ahmed et al. 2019). We extend this literature by documenting the general impact of manager characteristics on the properties of loan loss provisions (also in periods other than a crisis). Overall, this analysis suggests that the active intervention of individual managers in banks’ provisioning points to the opportunistic use of accounting discretion rather than to the use of superior private information.

Since the 2007–2008 financial crisis, bank governance has been an increasingly critical topic in important policy debates (e.g., Anginer et al. 2018; Becht et al. 2011; Fahlenbrach and Stulz 2011; Srivastav and Hagendorff 2016). Our findings inform these debates in at least two ways. First, the evidence that individual top management team members exert opportunistic influence supports recent regulatory efforts to constrain this influence on bank policies. Examples include the ECB’s implementation of fit and proper assessments for newly appointed top managers in systemically important banks as well as the M-component of U.S. supervisors’ CAMELS ratings. Second, the finding that effective bank governance can mitigate the adverse effects of managerial discretion on the informativeness of loan loss provisions underscores that supervisors should assess the characteristics of individual managers within the broader framework of the bank’s governance, rather than in isolation.

## Prior research and empirical predictions

### Individual managers and financial reporting choices

Economic theory offers ambiguous predictions on whether the individual characteristics of managers have any influence on corporate decisions. Neoclassical theory views managers as a homogeneous input of firms’ production processes (Bertrand and Schoar 2003; Veblen 1900). Relatedly, new institutional theory suggests that organizational boundaries, conventions, and norms constrain the impact of any individual on firm outcomes (e.g., DiMaggio and Powell 1983). In contrast to these predictions, Hambrick and Mason’s (1984) upper echelons theory argues managers’ personalities, experience, and values are the main drivers of the organizational decisions within a firm. Put differently, upper echelons theory suggests that two seemingly identical managers with similar levels of education, age, tenure, and compensation can vary in terms of how they affect corporate actions because of their latent unobservable personalities and abilities. More recent theory (e.g., Dessein and Santos 2020) attributes those idiosyncratic management styles to differences in attention allocation rather than to the cognitive biases of managers.

The accounting literature has tested these theories primarily in the context of financial reporting by nonfinancial firms. Overall, this evidence is consistent with both the upper echelons and attention theories. A first set of studies investigates the association between reporting practices and manager-specific variables, such as gender (Francis et al. 2015), age (Huang et al. 2012), tenure (Ali and Zhang 2015), masculinity (Jia et al. 2014), ability (Demerjian et al. 2013), cultural heritage (Brochet et al. 2019), and legal infractions (Davidson et al. 2015). While these studies demonstrate the existence of these associations, individual managerial traits are unlikely to manifest in isolation but rather in combinations of specific attributes (e.g., Adams et al. 2018). Therefore, a second set of studies explores whether general management styles that capture groups of many different latent individual traits influence reporting choices. The evidence suggests that those management styles help explain firms’ accrual quality (Dejong and Ling 2013; Ge et al. 2011; Wells 2020).

There is much less evidence for the banking industry and its particular accruals process, as this process is hardly comparable to that of other industries (Stubben 2010). While the literature suggests that individual loan officers affect loan terms and that manager styles are associated with risk-taking (e.g., Bushman et al. 2021; Costello et al. 2020; Herpfer 2021; Hagendorff et al. 2021), it remains unclear whether these findings extend to the influence of individual managers over loan loss provisioning.

### Individual managers and the informativeness of banks’ loan loss provisioning

To assess whether the influence of individual managers affects the informational properties of banks’ loan loss provisions, the first question is why managers would have such an influence in banking. One reason lies in the nature of a task as inherently subjective and complex as provisioning for future loan losses. In our sample period, banks must recognize loan loss provisions if it is probable that a loan is impaired and if the amount can be reasonably estimated. When bank managers assess these criteria, they frequently distinguish between general loan loss provisions for portfolios of homogeneous loans (e.g., different classes of consumer loans) and specific provisions for large individual loans. They use complex statistical models to estimate general loan loss provisions, and the input of these models is subject to substantial managerial judgment. The extent of this judgment is even greater when managers determine individual loan loss provisions for large commercial loans, and through these decisions, bank managers directly intervene in reporting decisions. While CEOs and CFOs are the most plausible candidates for these types of interventions, strong personalities on other positions, for example a CRO, could also have the means (e.g., Mikes 2009).

Another reason is the nature of banking as a highly regulated industry. On the one hand, regulation imposes strong constraints on individual managers, limiting their influence on such bank policies as provisioning (e.g., Beatty and Liao 2014; Hollander and Verriest 2016). On the other hand, bank regulators often prefer more conservative allowances than required by GAAP, which can give rise to even more management discretion.

If individual managers do affect banks’ financial reporting, the second question is whether their influence affects the informational properties of loan loss provisions and, if so, in which direction. Empirical predictions on this question are ambiguous. On the one hand, discretion can enhance transparency by allowing managers to convey private information (e.g., about future loan defaults) to external parties when they have superior knowledge about a transaction that otherwise cannot be reflected in the accounting system (e.g.,Beaver et al. 1989; Wahlen 1994). On the other hand, managers could exercise their discretion opportunistically to delay the recognition of losses, reducing the informativeness of the loan loss allowance about future losses and ultimately compromising bank transparency (e.g., Bischof et al. 2021; Vyas 2011). Therefore, it is ultimately an empirical question whether managerial discretion affects the informativeness of loan loss provisions.

### Interaction between corporate governance and manager discretion in banks’ loan loss provisioning

Bank managers do not act in isolation when deciding on corporate policies, such as loan loss provisioning. In each bank, governance mechanisms are supposed to align managers’ behavior with the interests of relevant bank stakeholders. These arrangements interfere with the incentives and abilities of individual managers to exert their personal management style. The impact is at least twofold. First, governance mechanisms limit the discretion available to managers. Second, governance constrains individual managers’ opportunistic use of their discretion.

Research on the relationship between corporate governance and accounting quality generally suggests that effective governance supports the monitoring of top management and thus helps limit accounting discretion (e.g., Anderson and Campbell 2004; Beasley 1996; Bourveau et al. 2018; Evans et al. 2025; Karamanou and Vafeas 2005; Zhang 2019). In particular, measures protecting the interests of shareholders, such as board independence (Chen et al. 2015; Coles et al. 2014) or low management inside ownership (Berger et al. 2016; Cheng et al. 2005), help protect shareholders against managerial opportunism. Similar evidence exists for internal governance measures related to the top management team, such as the diversity of demographic characteristics of appointed managers (Berger et al. 2014; Qi et al. 2018; Zhang et al. 2019). Governance can thus be a significant moderating factor in the relationship between manager styles and banks’ loan loss recognition (Anginer et al. 2018; Armstrong et al. 2010; Beatty and Liao 2014; Becht et al. 2011; Laeven and Levine 2009). If better-governed banks can limit the opportunism associated with individual manager discretion (e.g., Choi 2024), we would expect that effective governance would attenuate the negative association between the discretionary influence of individual managers over a bank’s loan loss provisioning and the informativeness of these provisions.

## Data

We combine bank financial accounting data from Compustat Banks and manager data from ExecuComp to estimate the effect of individual bank managers on loan loss provisions and other bank policies.^3^ To examine the economic consequences of this management discretion, we use stock market data from the Center for Research in Security Prices (CRSP). We describe the sample selection in Table 1. Our sample period spans from 1993 to 2021. Panel A presents the selection of the manager-year sample. We limit the dataset to 189 banks from the Compustat Banks file (representing 14,076 manager years) that employed at least one manager from the ExecuComp file who switched to another bank during the sample period, as this allows us to separate bank and manager effects with the AKM sampling technique. We then exclude 15 banks because of missing data on their loan loss provisions. Our final dataset, which contains available information on manager characteristics from BoardEx and ExecuComp and on bank characteristics from Compustat Banks, includes 13,906 manager-year observations and 2,201 managers from 174 banks. We exclude another 1,562 manager-year observations for the analyses that require data on banks’ regulatory capital and charge-offs (12,344 manager-years), 834 manager-year observations for the analyses that require data on nonperforming loans (11,510 manager-years), 874 manager-year observations because of missing data for the estimation of discretionary loan loss provisions (10,636 manager-years), and 265 manager-year observations because of missing data for the estimation of the timeliness of banks’ loan loss provisions (10,371 manager-years).

**Table 1:** Sample Selection

**Panel A: Manager-year Sample**		
	**#Observations**	**#Banks**
All executives in ExecuComp (1993–2021)	333,179	3,961
Intersection with Compustat Banks	20,339	280
*Thereof*: Manager-years in banks …		
(1) with at least one moving manager,	14,076	189
(2) without missing data on loan loss provisions (Table 4),	13,906	174
(3) without missing data on regulatory capital and charge-offs (Table 8),	12,344	164
(4) without missing data on nonperforming loans (Table 7 Panel A),	11,510	157
(5) without missing data to estimate discretionary LLPs (Table 6),	10,636	145
(6) without missing data for the estimation of LLP timeliness (Table 7 Panel B)	10,371	142
**Panel B: Bank-quarter Sample**		
	**#Observations**	**#Banks**
Compustat Banks (1993–2021)	92,223	2,176
*Thereof*: Bank-quarters …		
without duplicates,	92,201	2,176
without missing data on loan loss provisions or control variables,	39,902	2,176
without missing data over the 12-quarter rolling window,	32,103	1,045
with a bank-level match to the Manager-year sample used for Table 7 Panel B,	11,573	142
from only the fourth quarter	2,883	142

Table 1 presents sample selection. Panel A describes the manager-year sample for our main analysis of the influence of individual top managers over loan loss provisions. We start with all managers covered by ExecuComp between 1993 and 2021. We merge these data with Compustat Banks and delete all banks that did not employ at least one moving manager during our sample period and that have missing data on our main dependent variable (loan loss provisions). For additional analyses, we also delete banks with missing data variables on relevant independent variables (especially regulatory capital, charge-offs, and non-performing loans). Panel B describes the bank-quarter sample that we employ to calculate our *Timely LLP* measure for the analyses in Table 7 Panel B. Each line in the table shows the number of total observations and distinct banks that remain in the sample after the application of each filter

Following Wells (2020), we include all managers listed in ExecuComp, which generally encompasses the five highest-paid managers within each bank. We specifically identify top managers (executive suite level) based on their titles as listed in ExecuComp. In our banking setting, the classification includes the positions of CEO, CFO, CRO (chief risk officer), CIO (chief information officer), and COO (chief operating officer). In our final sample, 1,071 distinct managers hold these positions. While prior research documents that several top executives of banks, especially the CRO, are involved in the assessment of loan loss provisions (Mikes 2009), evidence from other industries implies that CEOs and CFOs differ in their influence on reporting decisions (Jiang et al. 2010). We further restrict the sample to the 844 distinct CEOs and CFOs in additional robustness tests to examine these potential differences.

Panel B presents the selection of the bank-quarter sample that we employ to estimate the timeliness of loan loss provisions at the bank level. We also derived this sample from Compustat Banks. After eliminating bank-quarters with missing data on loan loss provisions and independent variables necessary to estimate the model over the 12-quarter rolling window and after matching the sample to the banks included in our manager-year sample, we arrive at a sample of 11,573 bank-quarters and 142 distinct banks for this analysis. These bank-quarters represent 2,883 bank-years (i.e., observations from the fourth quarter) that we integrate into the manager-year sample.

## Individual manager styles and loan loss provisioning choices

In the first part of our analysis, we estimate the influence of individual managers on banks’ loan loss provisioning. We then compare the influence of individual managers over loan loss provisions to other bank policy choices.

### Research design

Banks and their executives are linked through contracts and incentives. Therefore, a methodological challenge is to separate manager fixed effects from the impact of bank characteristics on loan loss provisions. We rely on the AKM sampling technique to address this identification issue. This method allows us to distinguish between idiosyncratic bank influence and manager influence. Furthermore, we can partly avoid the identification issues that arise when a sample comprises only managers who switch employers at least once (as in Bertrand and Schoar 2003). Such a sample limitation would reduce our sample size and potentially introduce sample selection bias, especially if differences in the likelihood of managerial mobility correlate with loan loss provisioning. Nevertheless, we caution that top management changes are generally likely to be endogenous events. Therefore, it is possible that changes in leadership coincide with changes in provisioning policies or other major bank choices. While we further address these concerns by using plausibly exogenous turnovers in robustness tests, the limitation is inherent to any method that involves manager changes, irrespective of the sample size.

The AKM method exploits the interconnectedness of managers and banks within groups. Specifically, while the mover method can identify a manager effect only if the person was working for at least two banks throughout the sample period, the necessary and sufficient identifying condition of the AKM method is that a manager worked for a bank that employed at least one manager who went to work for another bank during the sample period (Abowd et al. 2002). Put differently, we can exploit information from all banks that employed at least one manager who switched employers at some point during the sample period. All other managers who worked for these banks can then also be included in our sample. Therefore, our sample includes a large proportion of nonmoving managers, reducing potential selection bias while increasing sample size. To obtain accurate estimates for the manager and bank fixed effects, the AKM method still requires a certain degree of mobility to avoid estimation bias (Andrews et al. 2008; Gormley and Matsa 2014). This limitation must be considered when interpreting the outcomes for both the mover method and the AKM method.^4^

**Fig. 1:**
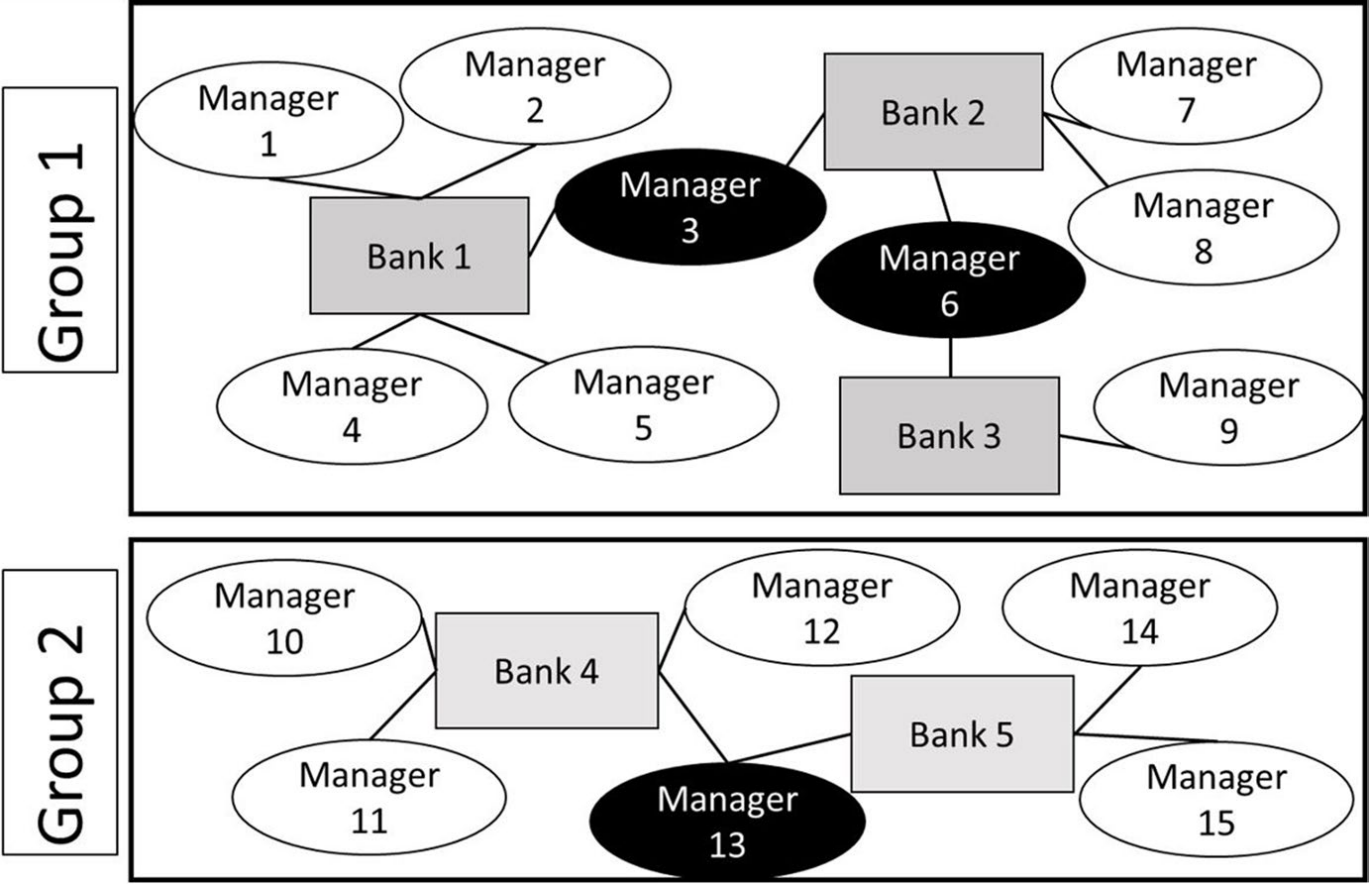
AKM Grouping Procedure connecting managers and banks within groups. Figure 1 shows the grouping procedure that allows to identify manager and firm fixed effects using the AKM (1999) sampling technique. The figure is adapted from Abowd, Creecy and Kramarz (2002). Within each group, all managers are connected to each other, while managers across groups are not connected. Managers who move between banks and thus establish the connectedness are highlighted in black

To form the groups of connected managers and banks, we follow the approach proposed by Abowd et al. (2002). We start with an arbitrarily chosen manager and include all banks this manager worked for during the sample period. In the second step, we include all managers who worked for these banks during this period. This procedure is repeated until we can add no more managers or banks to the group. We start over with the next group until all data are exploited. This algorithm results in groups of connected executives and banks that are connected within groups but not across groups. Abowd et al. (2002) formally prove that connectedness is necessary and sufficient for identification of worker and firm fixed effects. Figure 1 illustrates this grouping procedure.

Using the AKM method, we estimate the following three-way fixed effects model in our manager-year dataset (Table 1 Panel A) to specify the manager and bank effects on discretionary loan loss provisions:

1 $${LLP}_{\text{j}t}={{Bank\;FE}_{j}+Manager \;FE}_{i}+{Year\;FE}_{t}+\upvarepsilon ,$$

where $$i$$ denotes the executive,$$j$$ denotes the bank, and $$t$$ denotes the year. *LLP* is the ratio of bank j’s loan loss provisions in year t to beginning-of-the-year total loans. We include bank $$({Bank FE}_{j})$$, manager $$({Manager FE}_{i})$$, and year fixed effects ($${Year FE}_{t})$$. The main variable of interest is the manager fixed effects variable,$${Manager FE}_{i}$$, which captures time-invariant manager characteristics, such as gender, ability, and personality. Note that this model does not include time-varying manager or bank characteristics, as we are interested in the total effect of managers on loan loss provisions in the first step (Schoar et al. 2024; Hanlon 2022). If we controlled for time-varying bank attributes such as earnings, we would ignore the effect that managers have on loan loss provisions through earnings (management). We include time-varying bank controls in the second step when we explore how managers affect loan loss provisions. Because fixed effects are computed relative to a within-group benchmark, we normalize the fixed effects to allow comparisons across groups. Therefore, we follow the procedure recommended by Cornelissen (2008) and implemented by Graham et al. (2012) and Schoar et al. (2024) and normalize bank and manager fixed effects to have zero mean and unit variance.^5^ Note that negative values for the standardized manager fixed effect do not imply that the individual manager is associated with a decline in loan loss provisions. The negative (positive) values of the standardized variable rather represent a below-average (above-average) influence of the manager over total loan loss provisions; that is, the sign of the variable helps explain the magnitude, not the direction of the manager effect.

Once we have estimated the influence of individual managers over loan loss provisions, we compare this effect with other bank policy choices. To this end, we re-estimate Eq. (1) and replace LLP with proxies for those alternative bank policies as dependent variables. We employ *Bonus*, *Total Comp.*, and *Total Volatility* to capture managers’ intrinsic talents and risk preferences (Albuquerque et al. 2013; Francis et al. 2015; Graham et al. 2012; Schoar et al. 2024). Furthermore, we capture decisions related to loan portfolios with the loan-to-deposit ratio (*Loans/Deposits Ratio*), the ratio of nonperforming loans to gross loans (*NPL Ratio*), the ratio of loans to total assets (*Loan Ratio*), and banks’ non-interest income (*Non-Interest Income*). Banks with a higher proportion of loans naturally own fewer securities, and they typically follow a more traditional business model (Beltratti and Stulz 2012). Furthermore, *Loans/Deposits Ratio* captures whether loans are funded with deposits or other potentially riskier sources of funding (Laeven and Levine 2009). Finally, the *NPL ratio* captures the riskiness of banks’ past lending decisions (Bischof et al. 2022; Ghosh 2015).

### Results

Table 2 presents descriptive statistics on managerial mobility in our sample. To obtain accurate estimates of manager and bank fixed effects, a certain degree of mobility is necessary to avoid estimation bias (Andrews et al. 2008; Gormley and Matsa 2014). Descriptive statistics suggest that our sample is helpful in this regard. Relative to other studies, mobility appears to be high. Table 2 Panel A documents that 9.7% (214 out of 2,201) of the managers in our sample change employers at least once; the Graham et al. (2012) sample includes 4.9% movers. With the 214 moving managers, we can form 19 distinct groups composed of connected managers and banks. (See Table 2 Panel B for a detailed description of these group, and Fig. 1 for an illustration of the grouping procedure.) The largest connected group comprises 134 banks and 1,753 managers. These statistics illustrate that a relatively low share of managerial mobility still leads to a high degree of connectedness, which is one of the key advantages of the AKM method (Abowd et al. 2002).

**Table 2:** Manager mobility and connectedness

**Panel A: Number of movers out of all managers**				
**Mover**	**Number of firms in which managers have been employed**	**Number of managers**	**%**	**Cum.**
No	1	1,987	90.28	90.28
Yes	2	197	8.95	99.23
	3	13	0.59	99.82
	4	4	0.18	100
	Total	2,201	100	-
**Panel B: Groups of connected banks**				
**Group**	**Number of manager-years**	**Number of managers**	**Thereof: Moving managers**	**Number of banks**
1	47	14	1	2
2	10,974	1,753	189	134
3	296	40	1	2
4	67	16	1	2
5	100	13	1	2
6	231	34	2	3
7	252	40	1	2
8	170	31	1	2
9	162	21	3	3
10	77	17	1	2
11	80	12	1	2
12	172	28	1	2
13	207	33	2	3
14	193	18	2	2
15	223	37	1	2
16	65	14	1	2
17	122	23	2	2
18	297	39	2	3
19	171	18	1	2
Total	13,906	2,201	214	174
Thereof: C-Level Executives	6,478	1,071	85	114
Thereof: CEO/CFOs	5,377	844	68	100

Table 2 provides summary statistics about the mobility of managers in the sample. Panel A indicates how many managers moved between banks during our sample period. Panel B shows the groups formed using the AKM sampling technique (see Fig. 1) to identify the manager fixed effects for the connectedness sample. At least one moving manager connects all banks and managers within a certain group

Table 3 presents descriptive statistics for the manager characteristics (Panel A) and bank characteristics (Panel B) of our final sample. These statistics resemble those of other studies using bank managers as the unit of observation (e.g., Hagendorff et al. 2021). The average age of a bank manager in our sample is 54 years with an average tenure of 5.1 years. Approximately 7% of the managers in our sample are female. Panel A also reveals that the normalization of the manager fixed effects was successful and yields both a mean and a median of zero for this variable. In the interpretation of the manager fixed effects, note that manager fixed effects are estimated relative to a benchmark and require normalization to make them comparable across estimation groups.

**Table 3:** Summary statistics

**Panel A: Manager Characteristics**	**N**	**Mean**	**Sd**	**P25**	**Median**	**P75**	**P90**
Tenure	13,906	5.128	4.163	2.000	4.000	7.000	11.000
Salary	13,906	5.939	0.650	5.563	5.947	6.336	6.695
Bonus	13,998	3.066	2.860	0.000	3.907	5.525	6.621
Age	13,046	53.997	7.266	49.000	54.000	59.000	63.000
Total Compensation	12,759	7.122	1.067	6.354	7.011	7.792	8.611
Female	13,906	0.073	0.260	0.000	0.000	0.000	0.000
CEO	13,906	0.247	0.431	0.000	0.000	0.000	1.000
CFO	13,906	0.146	0.354	0.000	0.000	0.000	1.000
Other C-Level Executive	13,906	0.092	0.289	0.000	0.000	0.000	0.000
Highly Educated (MBA, PhD, CPA)	13,906	0.346	0.476	0.000	0.000	1.000	1.000
Manager Fixed Effects (after normalization)	13,906	0.000	0.012	−0.006	0.000	0.008	0.013
Manager Fixed Effects (before normalization)	13,906	0.012	0.012	0.005	0.011	0.020	0.024
**Panel B: Bank Characteristics**							
**Variable**	**N**	**Mean**	**SD**	**P25**	**Median**	**P75**	**P90**
LLP	13,906	0.007	0.014	0.002	0.004	0.008	0.017
NPL	13,312	0.017	0.048	0.006	0.009	0.018	0.033
ALL	13,906	0.017	0.010	0.011	0.014	0.019	0.028
Loan Growth	13,906	0.138	0.250	0.022	0.081	0.183	0.367
Loan Ratio	13,982	0.617	0.148	0.562	0.643	0.706	0.772
Size	13,906	9.838	1.517	8.720	9.631	10.720	11.868
ΔGDP	13,906	0.024	0.016	0.016	0.022	0.035	0.043
ΔCSRET	13,906	0.039	0.059	0.010	0.043	0.079	0.104
ΔUNEMP	13,906	0.015	0.208	−0.113	−0.036	0.018	0.256
CO	13,901	0.006	0.012	0.007	0.003	0.001	0.000
Loans/Deposits Ratio	13,965	0.872	0.224	0.778	0.884	0.979	1.110
EBP	11,735	0.038	0.065	0.022	0.030	0.041	0.056
Absolute DLLP	10,636	0.005	0.007	0.002	0.004	0.006	0.011
Non-interest Income	11,587	0.237	0.146	0.132	0.215	0.311	0.423
Tier 1	13,350	2.359	0.282	2.178	2.373	2.518	2.688
Total Volatility	7,753	8.611	5.521	5.216	7.043	10.185	14.661
High Governance Score (Raw)	9,012	9.974	2.843	8.000	10.000	12.000	14.000
Independent Board (Raw)	7,785	0.288	0.285	0.054	0.186	0.450	0.745
Low Inside Ownership (Raw)	10,515	0.646	2.537	0.078	0.184	0.531	1.210
High Gender Diversity (Raw)	12,344	0.111	0.161	0.000	0.000	0.278	0.320

Table 3 provides summary statistics. Panel A shows descriptive statistics for all manager variables. Panel B provides information about bank characteristics. All variables are defined in Appendix A. We show the raw values of high governance score, independent board, low inside ownership, high gender diversity before their median splits and show high gender diversity before the standardization to have a mean of zero

Table 4 presents the first part of our analysis. We estimate Eq. (1) to provide evidence on the influence of individual managers over a bank’s loan loss provisioning policies. We follow Wells (2020) and start with the Theil (1967) decomposition that illustrates the marginal explanatory power of including manager fixed effects to Eq. (1). Table 4 Panel A documents that the adjusted R^2^ increases between 3.29 percentage points (in the model with both year and bank fixed effects) and 16.40 percentage points (in the model with only year fixed effects) when adding manager fixed effects. This is an increase of the adjusted R^2^ by 9.36% or 76.39%, respectively. This increase is five times larger than the incremental R^2^ of adding bank fixed effects (0.57 percentage points = 38.44—37.87), indicating that the impact of manager fixed effects on loan loss provisions is economically meaningful. This increase is also comparable to that of prior studies (e.g., Wells 2020; Bushman et al. 2021) that document similar increases in explanatory power for firm-level choices, although it is considerably smaller than in studies that explore policy choices on the individual level, such as manager compensation (Graham et al. 2012) or inventor fixed effects (Liu et al. 2021).^6^ While this decomposition provides a first intuition consistent with the notion that individual manager fixed effects have a marginal impact on banks’ provisioning choices and thus serves as our starting point, it is not informative about the relative importance of these manager fixed effects compared to bank and year fixed effects.

**Table 4:** Economic and Statistical Significance of Manager Fixed Effects

**Panel A: Theil decomposition of the LLP variance explained by manager fixed effects**								
			**(1)**	**(2)**	**(3)**	**(4)**	**(5)**	**(6)**
Year Fixed Effects			Yes	Yes			Yes	Yes
Bank FE					Yes	Yes	Yes	Yes
**Manager Fixed Effects**				Yes		Yes		Yes
N = 13,906								
Testing Manager Fixed Effects = 0								
F-statistic				2.67		1.45		1.38
P-value				< 0.001		< 0.001		< 0.001
Identified Manager FE				2200		2200		2200
Adjusted R^2^			21.47%	37.87%	13.58%	18.83%	35.15%	38.44%
p.p. Increase in Adjusted R^2^				16.40%		5.25%		3.29%
% of comparable model				76.39%		38.66%		9.36%
**Panel B: Shapley Decomposition of Manager Fixed Effects**								
	**(1)**	**(2)**	**(3)**	**(4)**	**(5)**	**(6)**	**(7)**	**(8)**
	**LLP**	**Total Comp**	**Bonus**	**Loan Ratio**	**Loans/Deposits Ratio**	**NPL Ratio**	**Non-interest Income**	**Total Volatility**
Adj. total R^2^	38.44%	82.90%	65.85%	84.82%	82.95%	21.61%	87.85%	65.87%
Total R^2^	49.00%	86.09%	71.68%	87.41%	85.86%	35.43%	90.04%	72.30%
**Partial R**^**2**^** explained by manager FE**	**18.75%**	**37.35%**	**14.77%**	**14.38%**	**24.21%**	**25.35%**	**15.13%**	**2.34%**
% of Model adj. R^2^ explained by manager FE	48.78%	45.05%	22.43%	16.95%	29.19%	117.31%	17.22%	3.55%
Partial R^2^ explained by bank FE	8.24%	36.33%	17.87%	69.41%	54.88%	4.51%	67.62%	20.25%
% of adj. R^2^ explained by bank FE	21.44%	43.82%	27.14%	81.83%	66.16%	20.87%	76.97%	30.74%
Partial R^2^ Residual & Time Fixed Effects	73.01%	26.32%	67.35%	16.22%	20.91%	70.14%	17.25%	77.41%
*Statistical Significance of Fixed Effects (F-Tests)*								
Bank FE = 0	1.6939***	5.6845***	4.4822***	15.7599***	13.8627***	0.4095	21.4233***	4.5552***
Manager FE = 0	1.3248***	7.1815***	2.5628***	4.0338***	3.8216***	1.1237***	2.9017***	1.3550***
Manager and Bank FE = 0	2.6242***	25.335***	5.6284***	32.3386***	27.2398***	2.1763***	37.7171***	3.6596***
Number of Observations	13,906	12,759	13,998	13,982	13,965	13,312	11,587	7,753
Number of Identified Bank Effects	155	155	155	155	155	153	131	86
Number of Identified Manager Effects	2,200	2,201	2,205	2,205	2,203	2,166	1,932	1,355

Table 4 shows the economic and statistical importance of manager fixed effects. Panel A shows the adjusted R^2^ from the following regression:$LL{P}_{jt}=Bank Fixed Effects+Manager Fixed Effects+Year Fixed Effects+\varepsilon$. F-statistics indicate whether the manager fixed effects are jointly equal to 0. Panel B presents the Shapley decomposition of the variance explained by manager fixed effects, bank fixed effects, year fixed effects, and residuals when estimating Eq. (1) employing the AKM method. Partial R^2^ explained by manager fixed effects is the percentage of the variation in the dependent variable explained by the manager fixed effect. % of Model adj. R^2^ is the proportion of the model adj. R^2^ explained by the manager fixed effects. Statistical significance of the fixed effects is based on an F-test. The maximum number of banks is 174, with one bank serving as a reference bank in each AKM estimation group (174–19 = 155). Robust standard errors are clustered by bank. We define all variables in Appendix A. ***, **, and * indicate statistical significance at the 1%, 5%, and 10% levels (two-tailed), respectively

Therefore, we complement the results of the Theil decomposition with the Shapley decomposition, following Graham et al. (2012) and Wells (2020). The Shapley decomposition offers the advantage that we can estimate the economic importance of manager fixed effects relative to year and bank fixed effects; that is, the method provides the percentage of the adjusted R^2^ attributable to each type of fixed effect. Table 4 Panel B presents the results of the Shapley decomposition. Our results confirm the notion that individual managers can meaningfully influence a bank’s loan loss provisions. Column 1 shows that the 2,200 identified manager effects explain on average 18.75% of the variation in discretionary loan loss provisions, whereas bank-fixed effects explain only 8.24% of the variation (Column 1). The R^2^ decomposition thus indicates that idiosyncratic manager effects add explanatory power to loan loss provision models that is twice as large as the relative explanatory power of bank fixed effects.

To add some context, the average loan loss provision in our sample corresponds to 0.7% of the banks’ total assets. The average fraction of discretionary provisions lies in the range between 31.1% and 73.8% of total provisions, that is, between 0.21% and 0.52% of total assets (depending on the estimation model). Our results suggest that a manager with an above-average influence on loan loss provisions is associated with an increase in discretionary loan loss provisions by 0.4 percentage points; that is, the discretionary provisions increase from 0.21% to 0.61% (at the lower bound of the estimate for discretionary accruals) or from 0.52% to 0.92% of total assets (at the upper bound). The magnitude of the difference between a low-influence and a high-influence manager thus appears economically meaningful.

To compare managers’ loan loss provisioning styles with their idiosyncratic influence on other bank policy choices, we rerun our analysis with other bank policy choices as our dependent variable. In all the specifications (presented in Columns 2 to 8 of Table 4, Panel B), we document that manager fixed effects explain between 2.34% (for volatility as an overall measure for risk policies) and 37.35% (for compensation policies) of the total variation in the different policy choices. As expected, the impact of individual managers on bank policies is thus not unique to the accounting choice. In particular, manager fixed effects matter more for compensation (*Total Compensation*) and loan portfolio decisions (*NPL Ratio* and *Loan/Deposit Ratio*) than for loan loss provisions. In particular, the findings regarding compensation policies very much comport with those of Graham et al. (2012) regarding nonfinancial industries. Panel B further provides F-statistics for manager fixed effects, bank fixed effects, and a combination of both. We find, for all accounting and policy choices, that manager and bank fixed effects are individually and jointly significantly different from zero at the 1 percent level (p-value < 0.01), except for the *NPL Ratio* where we only document significant manager and combined manager and bank fixed effects.

While the AKM method serves to disentangle manager effects from bank effects for a given decision and thus helps us attribute a bank’s overall provisioning policy to the influence of an individual manager, it does not help address the overlap with other types of decisions over which the same manager also exerts individual influence. To provide initial evidence on this question, Table 5 reports the pairwise correlations between the manager fixed effects for the different bank policies. These correlations document that loan loss provisioning styles are highly interrelated with managers’ idiosyncratic preferences regarding other policy choices. Thus, a manager sets the personal provisioning style in a bundle with other management decisions, especially those for compensation, lending, and risk-taking. Put differently, a manager for whom the AKM method identifies a significant individual influence over a bank’s loan loss provisions appears to influence these other bank policies in a similarly individual way.

**Table 5:** Pairwise Correlation of Management Styles

	LLP	Total Compensation	Bonus	Loan Ratio	Loan/Deposit Ratio	NPL Ratio	Non-interest Income	Total Volatility
LLP	1.000							
Total Compensation	0.189	1.000						
	(0.000)							
Bonus	0.132	0.167	1.000					
	(0.000)	(0.000)						
Loan Ratio	0.126	−0.224	−0.183	1.000				
	(0.000)	(0.000)	(0.000)					
Loans/Deposits Ratio	0.331	0.209	−0.063	0.602	1.000			
	(0.000)	(0.000)	(0.000)	(0.000)				
NPL Ratio	0.128	−0.048	−0.017	0.020	−0.106	1.000		
	(0.000)	(0.000)	(0.141)	(0.094)	(0.000)			
Non-Interest Income	0.313	0.007	0.165	−0.038	−0.000	0.050	1.000	
	(0.000)	(0.527)	(0.000)	(0.001)	(0.981)	(0.000)		
Total Volatility	0.598	0.127	−0.117	0.136	0.126	0.322	0.332	1.000
	(0.000)	(0.000)	(0.000)	(0.000)	(0.000)	(0.000)	(0.000)	

Table 5 shows pairwise correlations between manager fixed effects for eight different policy choices from Table 4, Panel B (with p-values in brackets). We define all variables in Appendix A

## Consequences of managers’ loan loss provisioning styles

The second part of our analysis relates the influence of individual managers to the consequences of banks’ loan loss provisioning. We use the estimated manager fixed effects from Eq. (1) to examine the association between managers’ loan loss provisioning styles and three outcomes: (i) the magnitude of absolute discretionary provisions, (ii) the timeliness of the provisions, and (iii) the mapping of the allowance for credit losses into future charge-offs.

### Discretionary loan loss provisions

#### Research design

We first estimate absolute discretionary accruals at the manager-year level (Table 1 Panel A) employing Beatty and Liao’s (2014) preferred model^7^:

2 $${LLP}_{jt}={\upbeta }_{0}+{\upbeta }_{1}\Delta {NPL}_{jt+1}+{\upbeta }_{2}\Delta {NPL}_{jt}+{\upbeta }_{3}\Delta {NPL}_{jt-1}+{\upbeta }_{4 }\Delta {NPL}_{jt-2}+{\upbeta }_{5 }{Size}_{jt-1}+{\upbeta }_{6}{\Delta Loan}_{jt}+{\upbeta }_{7}{\Delta GDP}_{t}+{\upbeta }_{8}{\Delta CSRET}_{t} +{\upbeta }_{9}{\Delta UNEMP}_{t}+\upvarepsilon ,$$

where *LLP* are loan loss provisions scaled by lagged total loans.$\Delta {NPL}_{t}$ is the change in nonperforming loans from period t-1 to t scaled by lagged total loans. $\Delta {NPL}_{t+1}, \Delta {NPL}_{t-1}$, and $\Delta {NPL}_{t-2}$ are defined accordingly and are intended to capture changes in provisioning due to changes in loan portfolio quality. Size is the natural logarithm of total assets. Δ*GDP* is the change in GDP over the year. Δ*CSRET* is the return on the Case-Shiller Real Estate Index over the year. Δ*UNEMP* is the change in unemployment rates over the quarter. Both macro variables are obtained from the Federal Reserve Bank of St. Louis. Following Beatty and Liao (2011) and Nicoletti (2018), we use the average absolute residuals from estimating Eq. (2) as our measure for discretionary provisioning (*Absolute DLLP*).

We then test whether managers’ idiosyncratic provisioning styles correlate with the magnitude of a bank’s discretionary loan loss provisions. Therefore, we use our manager-year sample (Table 1 Panel A) to regress the proxy for discretionary provisioning from Eq. (2) on four different variables that capture managers’ loan loss provisioning preferences:

3 $${Absolute \;DLLP}_{jt}={\upbeta }_{0}+{\upbeta }_{1}{Manager\; LLP \;Style}_{i}+ {\upbeta }_{2}\Delta {NPL}_{jt+1}+{\upbeta }_{3}\Delta {NPL}_{jt}+{\upbeta }_{4}\Delta {NPL}_{jt-1}+{\upbeta }_{5}\Delta {NPL}_{jt-2}+ {\upbeta }_{6 }{EBP}_{jt}+{\upbeta }_{7 }{Size}_{jt-1}{+ {\upbeta }_{8 }{Tier\; 1 \;Capital}_{jt-1}+\upbeta }_{9}{\Delta Loan}_{jt} +{Bank \;FE}_{j}+{Year FE}_{t}+\upvarepsilon ,$$

where *Absolute DLLP* are the absolute residuals estimated in Eq. (2). We employ four proxies for *Manager LLP Style* that all build on the manager fixed effects estimated in Eq. (1). First, we use the raw (continuous) fixed effect estimated in Eq. (1) normalized according to Cornelissen (2008). Second, we employ a binary variable that takes the value of 1 if the normalized manager fixed effect exceeds 0 and 0 otherwise (*High Discretion Manager*). Third, we use a binary variable that takes the value of 1 if the normalized manager fixed effect is in the highest quartile of the variable’s distribution and 0 otherwise (*Highest Discretion Manager*). These three proxies are all expected to be positively associated with the fraction of a bank’s total loan loss provisions that is captured by the individual manager’s fixed effect in Eq. (1), that is, an indicator of an individual manager’s influence over the bank’s total loan loss provisions. Fourth, we use a binary variable that takes the value of 1 if the normalized manager fixed effect is in the lowest quartile of the variable’s distribution and 0 otherwise (*Lowest Discretion Manager*). This proxy is expected to be negatively associated with the individual manager’s influence over a bank’s loan loss provisions. We capture changes in abnormal provisions due to earnings or capital management incentives with additional controls for *EBP* (earnings before loan loss provisions) and *Tier 1* capital. *EBP* is pre-tax income before provisions divided by beginning of the year total loans. *Tier 1* is the natural logarithm of banks’ Tier 1 capital ratio measured at the beginning of the period. We define all other variables as in Eq. (2). To account for a potential correlation in the error terms due to the inclusion of multiple managers per bank, we cluster standard errors on the bank level.

#### Results

Our results in Table 6 show that the influence of individual managers over loan loss provisions is significantly positively associated with banks’ absolute discretionary loan loss provisions. The coefficient estimates for the continuous measure and the two binary variables (*High Discretion Managers* and *Highest Discretion Managers*) are significantly positive, while the coefficient estimate for the *Lowest Discretion Managers* is significantly negative. This indicates that the greater the influence of individual managers, the higher the magnitude of a bank’s absolute discretionary loan loss provisions; that is, managers who actively influence banks’ accounting choices tend to increase the discretionary portion of the banks’ loss provisions.

**Table 6:** Manager Discretion and Discretionary Loan Loss Provisions

	(1)	(2)	(3)	(4)
*Manager LLP Style:*	Manager Fixed Effect (continuous)	High Discretion Manager	Highest Discretion Manager	Lowest Discretion Manager
*Dependent Variable:*	Absolute DLLP	Absolute DLLP	Absolute DLLP	Absolute DLLP
Manager LLP Style	0.427***	0.004***	0.005***	−0.003**
	(0.001)	(0.003)	(0.000)	(0.033)
ΔNPL _t+1_	−0.071***	−0.076***	−0.076***	−0.075***
	(0.000)	(0.000)	(0.000)	(0.000)
ΔNPL _t_	−0.039	−0.030	−0.031	−0.030
	(0.174)	(0.332)	(0.317)	(0.338)
ΔNPL _t-1_	0.040	0.051*	0.052*	0.052*
	(0.113)	(0.066)	(0.058)	(0.064)
ΔNPL _t-2_	−0.001	0.005	0.007	0.008
	(0.974)	(0.845)	(0.777)	(0.735)
EBP	0.005	0.006	0.007	0.006
	(0.495)	(0.487)	(0.463)	(0.506)
Tier 1	0.000	0.000	0.000	0.000
	(0.836)	(0.944)	(0.837)	(0.957)
Size	−0.001	−0.000	−0.000	−0.000
	(0.221)	(0.470)	(0.454)	(0.675)
Loan Growth	0.000	0.000	0.000	0.000
	(0.684)	(0.774)	(0.808)	(0.749)
Constant	0.010*	0.006	0.007	0.008
	(0.092)	(0.316)	(0.293)	(0.252)
N	10,636	10,636	10,636	10,636
Adj. R^2^	0.436	0.379	0.386	0.371
Bank and Year FE	Yes	Yes	Yes	Yes

Table 6 shows regressions of Absolute DLLP on binary variables for the different Manager LLP Styles that are determined from the manager fixed effects estimated in Table 4 Panel B. First, we use the raw (continuous) fixed effect estimated in Eq. (1). Second, we employ a binary variable that takes the value of 1 if the manager fixed effect is above 0 and 0 otherwise (High Discretion). Third, we use a binary variable that takes the value of 1 if the manager fixed effect is in the highest quartile of the manager fixed-effect distribution, and 0 otherwise (Highest Discretion Manager). Fourth, we use a binary variable that takes the value of 1 if the manager fixed effect is in the lowest quartile of the distribution, and 0 otherwise (Lowest Discretion Manager). All other variables are defined in Appendix A. P-values are based on robust standard errors clustered by bank. ***, **, and * indicate statistical significance at the 1%, 5%, and 10% levels (two-tailed), respectively

### Loan loss provision timeliness

#### Research design

We use two different models from the literature to examine the effect of managerial influence on the timeliness of loan loss provisions. The first model is frequently used to measure provision timeliness (e.g., Nicoletti 2018) and is motivated by the model that performs best in Beatty and Liao’s (2014) analysis. We add bank and time fixed effects to this model and use the manager-year sample (Table 1 Panel A) to estimate the timeliness of a bank’s loan loss provisions as follows:

4 $${LLP}_{jt}={\upbeta }_{0}+ {\upbeta }_{1}\Delta {NPL}_{jt+1}+{\upbeta }_{2}Manager\; {LLP\; Style}_{i}+{{\upbeta }_{3}\Delta {NPL}_{jt+1}* Manager\; {LLP \;Style}_{i}+\upbeta }_{4}\Delta {NPL}_{jt}+{\upbeta }_{5}\Delta {NPL}_{jt}* {Manager \;LLP \;Style}_{i}+{\upbeta }_{6}\Delta {NPL}_{jt-1}+{\upbeta }_{7}\Delta {NPL}_{jt-1}* Manager {LLP Style}_{i}+{\upbeta }_{8}\Delta {NPL}_{jt-2}+{\upbeta }_{9}\Delta {NPL}_{jt-2}* {Manager \;LLP \;Style}_{i}+{\upbeta }_{10}{Size}_{jt-1}{+\upbeta }_{11}{\Delta \;Loan}_{jt} +{Bank FE}_{j}+{Year \;FE}_{t}+\upvarepsilon ,$$

where all control variables are as defined in Eqs. (2) and (3). Our main coefficient of interest is ${\upbeta }_{3}$ and captures the incremental association between a specific loan loss provisioning style and the incorporation of forward-looking changes in nonperforming loans into loan loss provisions. We cluster the robust standard errors by bank.

We employ a second model for loan loss provision timeliness following Beatty and Liao (2011) and Bushman and Williams (2015).^8^ We use the bank-quarter sample (Table 1 Panel B) to measure loan loss provision timeliness as the difference in the R^2^ between Eqs. (5) and (6) using a 12-quarter rolling window, requiring banks to have data for all 12 quarters.

5 $${LLP}_{jt}={\upbeta }_{0} + {\upbeta }_{1}\Delta {NPL}_{jt+1}+{\upbeta }_{2}\Delta {NPL}_{jt}+{\upbeta }_{3}\Delta {NPL}_{jt-1}+{\upbeta }_{4}\Delta {NPL}_{jt-2}+ {\upbeta }_{5 }{EBP}_{jt}+{\upbeta }_{6 }{Size}_{jt-1}+ {\upbeta }_{7 }{Tier \;1 \;Capital}_{jt-1}+\upvarepsilon ;$$

6 $${LLP}_{jt}={\upbeta }_{0} + {\upbeta }_{1}\Delta {NPL}_{jt-1}+{\upbeta }_{2}\Delta {NPL}_{jt-2}+{\upbeta }_{3 }{EBP}_{jt}+{\upbeta }_{4 }{Size}_{jt-1}+ {\upbeta }_{5 }{Tier\; 1\; Capital}_{jt-1}+\upvarepsilon .$$

All variables are defined as in Eqs. (2) and (3). We subtract the R^2^ of Eq. (6) from that of Eq. (5) to yield an incremental R^2^. A higher incremental R^2^ reflects a timelier recognition of loan losses, and we expect larger incremental explanatory power as more information about future NPLs is conveyed by the current LLPs (i.e., the R^2^ from equation [5] to exceed that from equation [6]). In each quarter of our sample period, we rank banks on their incremental R^2^ and construct a binary variable *Timely LLP* that takes the value of 1 if the bank is above the median incremental R^2^ for more than two quarters of the year and 0 otherwise.

We then include the *Timely LLP* variable in our manager-year sample (Table 1 Panel A) to analyze the relationship between individual manager discretion and the timeliness of banks’ loan loss provisions. We estimate the following equation:

7 $${Timely\; LLP}_{jt}={\upbeta }_{0} +{\upbeta }_{1}Manager\; {LLP \;Style}_{i}+ {\upbeta }_{2}\Delta {NPL}_{jt+1}+{\upbeta }_{3}\Delta {NPL}_{jt}+{\upbeta }_{4}\Delta {NPL}_{jt-1}+{\upbeta }_{5}\Delta {NPL}_{jt-2}+ {\upbeta }_{6 }{EBP}_{jt}+{\upbeta }_{7}{Size}_{jt-1}+ {\upbeta }_{8}{Tier \;1 \;Capital}_{jt-1}+{State FE}_{c}+{Year FE}_{t}+\upvarepsilon .$$

All variables in Eq. (7) are defined as in Eqs. (2) and (3). We use state fixed effects for mainly two reasons. First, the measure of timeliness is a binary variable that naturally limits the variation of the timeliness of loan loss provisions within a bank. Second, when using bank-fixed effects, we would also need a within-bank change in management for the identification of the *Manager LLP Style* variable. Because of this lack of within-bank variation, we follow the literature and use alternative fixed-effects models. Specifically, we borrow the model with state fixed effects from Nicoletti (2018) and test the robustness of this research design choice. Other studies use no fixed effects (Beatty and Liao 2011), year fixed effects (Bushman and Williams 2015), or region and year fixed effects (Wheeler 2019; Gallemore 2023). Again, we cluster the robust standard errors by bank.

#### Results

Table 7 presents the results for the impact of managers’ loan loss provisioning styles on the timeliness of provisions. Table 7 Panel A shows that *High Discretion Managers* and *Highest Discretion Managers* incorporate forward-looking information (about future NPLs) to a lesser extent into current loan loss provisions than low-discretion managers (i.e., the coefficient estimate for the interaction of the manager style measure and the forward-looking Δ*NPL*_t+1_ [backward-looking Δ*NPL*_t-1_] is significantly positive [negative]). We corroborate this finding with our second timeliness measure in Panel B of Table 7. Controlling for bank characteristics and changes in nonperforming loans, we find that *High Discretion Managers* and *Highest Discretion Managers* are associated with a 5.1% and a 5.6% lower likelihood of a bank being classified as a *Timely LLP* bank, respectively (p-values < 0.01).

**Table 7:** Manager Discretion and Provision Timeliness

**Panel A: Manager Discretion and the Incorporation of Future Changes in NPLs into Loan Loss Provisions**						
	**(1)**	**(2)**	**(3)**	**(4)**	**(5)**	**(6)**
***Manager LLP Style:***	***High Discretion Manager***		***Highest Discretion Manager***		***Lowest Discretion Manager***	
***Dependent Variable:***	**LLP**	**LLP**	**LLP**	**LLP**	**LLP**	**LLP**
Manager LLP Style	0.003***	0.007***	0.003***	0.007***	−0.001*	−0.006***
	(0.000)	(0.000)	(0.000)	(0.000)	(0.054)	(0.000)
ΔNPL _t+1_	−0.008	−0.003	−0.004	0.002	−0.125***	−0.108***
	(0.643)	(0.852)	(0.867)	(0.900)	(0.000)	(0.000)
Manager LLP Style * ΔNPL _t+1_	−0.102***	−0.082***	−0.104***	−0.088**	0.125***	0.111***
	(0.002)	(0.009)	(0.006)	(0.015)	(0.000)	(0.000)
ΔNPL _t_	0.186***	0.190***	0.164***	0.170***	0.218***	0.178***
	(0.000)	(0.000)	(0.000)	(0.000)	(0.000)	(0.000)
Manager LLP Style * ΔNPL _t_	0.019	−0.015	0.067	0.032	−0.035	0.010
	(0.651)	(0.747)	(0.131)	(0.495)	(0.419)	(0.840)
ΔNPL _t-1_	0.034	0.031	0.073	0.062	0.255***	0.198***
	(0.446)	(0.402)	(0.181)	(0.158)	(0.000)	(0.000)
Manager LLP Style * ΔNPL _t-1_	0.238***	0.183***	0.180**	0.128*	−0.227***	−0.174***
	(0.001)	(0.004)	(0.038)	(0.076)	(0.001)	(0.005)
ΔNPL _t-2_	0.090***	0.060***	0.108***	0.080***	0.101***	0.075***
	(0.007)	(0.009)	(0.000)	(0.001)	(0.000)	(0.004)
Manager LLP Style * ΔNPL _t-2_	0.022	0.015	0.027	−0.005	−0.004	−0.006
	(0.584)	(0.607)	(0.568)	(0.895)	(0.924)	(0.850)
Size	0.001***	0.000	0.001***	0.000	0.001***	0.000
	(0.005)	(0.730)	(0.005)	(0.712)	(0.005)	(0.467)
Loan Growth	0.000	0.001	0.000	0.000	0.000	0.001
	(0.802)	(0.613)	(0.828)	(0.718)	(0.859)	(0.555)
ΔGDP	−0.065***		−0.068***		−0.064***	
	(0.000)		(0.000)		(0.000)	
CSR	−0.057***		−0.057***		−0.056***	
	(0.000)		(0.000)		(0.000)	
ΔUnemployment	−0.001		−0.000		−0.001	
	(0.374)		(0.771)		(0.161)	
Constant	0.003	0.002	0.004	0.003	0.005**	0.004
	(0.164)	(0.753)	(0.106)	(0.579)	(0.039)	(0.409)
N	11,510	11,510	11,510	11,510	11,510	11,510
Adj. R^2^	0.490	0.674	0.475	0.666	0.481	0.671
Bank and Year FE	No	Yes	No	Yes	No	Yes
**Panel B: Manager Discretion and Provision Timeliness**						
	**(1)**	**(2)**	**(3)**	**(4)**	**(5)**	**(6)**
***Manager LLP Style:***	***High Discretion Manager***		***Highest Discretion Manager***		***Lowest Discretion Manager***	
***Dependent Variable:***	**Timely LLP**	**Timely LLP**	**Timely LLP**	**Timely LLP**	**Timely LLP**	**Timely LLP**
Manager LLP Style	−0.073**	−0.071**	−0.058*	−0.061**	0.063**	0.059**
	(0.029)	(0.026)	(0.072)	(0.045)	(0.035)	(0.046)
Size		0.016*		0.017*		0.016*
		(0.095)		(0.071)		(0.094)
EBP		−0.148		−0.188		−0.178
		(0.174)		(0.104)		(0.120)
Tier 1		−0.060		−0.061		−0.054
		(0.308)		(0.297)		(0.363)
ΔNPL _t+1_		−0.367		−0.391		−0.351
		(0.563)		(0.538)		(0.583)
ΔNPL _t_		−1.802		−1.812		−1.810
		(0.131)		(0.129)		(0.129)
ΔNPL _t-1_		−0.374		−0.405		−0.385
		(0.742)		(0.720)		(0.734)
ΔNPL _t-2_		−0.409		−0.454		−0.452
		(0.675)		(0.637)		(0.642)
Constant	0.361***	0.352**	0.340***	0.329*	0.310***	0.295*
	(0.000)	(0.041)	(0.000)	(0.053)	(0.000)	(0.094)
N	10,371	10,371	10,371	10,371	10,371	10,371
Adj. R^2^	0.077	0.081	0.075	0.080	0.076	0.080
State and Year FE	Yes	Yes	Yes	Yes	Yes	Yes

Table 7 Panel A presents the results of estimating Eq. (4), which examines the association between a bank’s loan loss provision timeliness and managers’ loan loss provisioning styles. Manager LLP Styles are determined from the manager fixed effects estimated in Table 4 Panel B. First, we employ a binary variable that takes the value of 1 if the manager fixed effect is above 0 and 0 otherwise (High Discretion). Second, we use a binary variable that takes the value of 1 if the manager fixed effect is in the highest quartile of the manager fixed-effect distribution and 0 otherwise (Highest Discretion Manager). Third, we use a binary variable that takes the value of 1 if the manager fixed effect is in the lowest quartile of the distribution and 0 otherwise (Lowest Discretion Manager). Panel B presents the results of estimating Eq. (7), which examines the same association between loan loss provision timeliness and managers’ loan loss provisioning styles using an alternative measure for provision timeliness. Timely LLP is a binary indicator variable that takes the value of 1 if a bank’s delayed expected loss recognition estimated in Eqs. (5) and (6) is below the sample median and 0 otherwise. We define Size, EBP, and Tier 1 in Appendix A. P-values are based on robust standard errors clustered by bank. ***, **, and * indicate statistical significance at the 1%, 5%, and 10% levels (two-tailed), respectively

### Mapping of the allowance for credit losses into future charge-offs

#### Research design

In the next step, we explore whether the discretion employed by managers has consequences for the informativeness of the allowance about future loan charge-offs. Therefore, we use the manager-year sample (Table 1 Panel A) to estimate the following model based on Beck and Narayanamoorthy (2013) and Altamuro and Beatty (2010):

8 $${Charge-offs}_{jt+1}={\upbeta }_{0}+ {\upbeta }_{1}{ALL}_{jt}+{\upbeta }_{2}{Manager \;LLP \;Style}_{i}+{\upbeta }_{3} {ALL}_{jt}* {Manager LLP Style}_{i}+{\upbeta }_{4 }{Size}_{jt-1}{+\;{\upbeta }_{5 }{Tier \;1 \;Capital}_{jt-1}+\upbeta }_{6}{\Delta Loan}_{jt-1} +{Bank FE}_{j}+{Year FE}_{\text{t}}+\upvarepsilon ,$$

where we measure *Charge-offs* of bank j in year t + 1 (and, in an alternative specification, in year t + 2) and scale them by lagged total loans (i.e., measured at the beginning of the respective year). *ALL* is the allowance for loan losses divided by total loans. All other variables are defined as in Eqs. (2) and (3), and we cluster the robust standard errors by bank.

#### Results

Table 8 presents our analysis of whether banks’ loan loss allowances are also less informative about future realizations of loan losses when individual managers influence the loan loss provisions. The findings go in the same direction as the timeliness analysis. Columns 1 to 4 show that the primary coefficient of interest for the interaction of manager discretion with the allowance for loan losses is significantly negative, using loan charge-offs for one or two years ahead as the dependent variable. Consistently, we document in Columns 5 and 6 a lower association between the current loss allowance and future loan charge-offs for managers with the lowest discretion. Overall, these results are consistent with a decrease in the informativeness of a bank’s loan loss provisioning when individual managers actively influence the underlying accounting policies.

**Table 8:** Manager Discretion and Loan Loss Provision Informativeness about Future Charge-Offs

	(1)	(2)	(3)	(4)	(5)	(6)
*Manager LLP Style:*	*High Discretion Manager*		*Highest Discretion Manager*		*Lowest Discretion Manager*	
*Dependent Variable:*	CO _t+1_	CO _t+2_	CO _t+1_	CO _t+2_	CO _t+1_	CO _t+2_
Manager LLP Style * ALL	−0.164**	−0.072	−0.119*	−0.049	0.167**	0.043
	(0.019)	(0.181)	(0.057)	(0.247)	(0.047)	(0.525)
Manager LLP Style	0.006***	0.005***	0.006***	0.005***	−0.007***	−0.005***
	(0.000)	(0.000)	(0.000)	(0.000)	(0.000)	(0.000)
ALL	0.565***	0.208***	0.512***	0.186***	0.417***	0.152***
	(0.000)	(0.000)	(0.000)	(0.000)	(0.000)	(0.001)
Size	0.001***	0.002***	0.001***	0.002***	0.002***	0.002***
	(0.006)	(0.005)	(0.005)	(0.005)	(0.002)	(0.002)
Tier 1	−0.004***	−0.003*	−0.004***	−0.003*	−0.004***	−0.003**
	(0.002)	(0.052)	(0.003)	(0.069)	(0.001)	(0.039)
Loan Growth	0.001	0.001	0.001	0.001	0.001	0.001
	(0.198)	(0.366)	(0.203)	(0.371)	(0.222)	(0.409)
Constant	−0.009	−0.013	−0.008	−0.012	−0.006	−0.010
	(0.151)	(0.162)	(0.203)	(0.194)	(0.333)	(0.253)
N	12,344	11,479	12,344	11,479	12,344	11,479
Adj. R^2^	0.643	0.571	0.640	0.569	0.642	0.571
Bank and Year FE	Yes	Yes	Yes	Yes	Yes	Yes

Table 8 shows regression results for the differential effect of managers’ provisioning styles on the predictive value of banks’ loan loss allowances for realized loan charge-offs one year ahead (CO_t+1_) or two years ahead (CO_t+2_). Manager LLP Styles are determined from the manager fixed effects estimated in Table 4 Panel B: First, we employ a binary variable that takes the value of 1 if the manager fixed effect is above 0 and 0 otherwise (High Discretion). Second, we use a binary variable that takes the value of 1 if the manager fixed effect is in the highest quartile of the manager fixed-effect distribution and 0 otherwise (Highest Discretion Manager). Third, we use a binary variable that takes the value of 1 if the manager fixed effect is in the lowest quartile of the distribution and 0 otherwise (Lowest Discretion Manager). We define ALL, Size, Tier 1, and Loan Growth in Appendix A. P-values are based on robust standard errors clustered by bank. ***, **, and * indicate statistical significance at the 1%, 5%, and 10% levels (two-tailed), respectively

## The moderating effect of a bank’s corporate governance

### Research design

We continue our analysis by exploring the moderating role of a bank’s governance in the relationship between the influence of individual managers over a bank’s loan loss provisions and the informativeness of these loan loss provisions. We employ four proxies for bank governance from the literature.

The first three proxies capture measures that directly aim to protect shareholders. First, we use the score constructed by Gompers et al. (2003), which combines 24 different mechanisms potentially built into a bank’s governance to restrict shareholder influence. We obtain the data on these shareholder-unfriendly provisions from the Investor Responsibility Research Center (IRRC) Governance Legacy Dataset. *High Governance Score* takes the value of 1 if the index (i.e., the count of all shareholder-unfriendly governance provisions identified for a bank) is below the sample median in 2006.^9^ Second, we employ a measure of board independence from Coles et al. (2014). It captures the proportion of directors appointed after the current CEO has taken office. Following Coles et al. (2014), we assume that directors whom the CEO appoints become less independent and increase their influence over time. Therefore, we define independence as the sum of the tenure of directors appointed after the CEO scaled by the total tenure of all directors. Our proxy *Independent Board* takes the value of 1 if this tenure-weighted proportion of directors who have assumed office after the CEO is equal to or below the yearly median. Our third measure captures the proportion of inside stock ownership by the top management team, based on data obtained from ExecuComp. The literature documents that higher ownership stakes induce greater risk-taking by managers due to moral hazard issues (Berger et al. 2016). *Low Inside Ownership* takes the value of 1 if the bank’s average top management team ownership percentage is below the median during the sample period.

The last measure captures top management gender diversity as another dimension of bank governance (Qi et al. 2018; Zhang 2019).We start by estimating the Blau (1977) index that measures the homogeneity of a management team. The index is equal to the sum of the squared proportions of managers in each of the categories of the respective variable; that is, we define diversity as $1- {\sum }_{i=1}^{N}{p}_{i}^{2}$, where p is the proportion of managers in one of the two categories of gender (female, male). For example, the Blau index takes a value of 1 – (0.5^2^ + 0.5^2^) = 0.5 for a perfectly gender-balanced team with 50% female and male managers, whereas an all-male team receives a score of 0 (= 1–1^2^). Following Zhang (2019), we standardize the Blau index to have a mean of zero by first subtracting the sample mean and then dividing it by the standard deviation. *High Gender Diversity* takes a value of 1 if the standardized Blau index for the bank year exceeds or equals to the yearly median.

To analyze the interaction of our governance measures with a manager’s individual influence on the informativeness of loan loss provisions, we include the interaction term between *High Discretion* and each of the binary governance variables in Eq. (8).^10^

### Results

Table 9 reports the results of our analysis of the interplay between individual management styles and four different moderators related to a bank’s corporate governance. We document that all four proxies for effective governance (shareholder rights, board independence, low inside ownership, gender diversity) significantly mute the negative association between the high influence of individual managers over loan loss provisions (*High Discretion*) and the predictability of future loan charge-offs. This pattern is broadly consistent with the notion that specific governance mechanisms can help limit the extent to which individual managers can opportunistically use their reporting discretion in recognizing loan loss provisions.

**Table 9:** Bank Governance, Manager Discretion and Loan Loss Provision Informativeness about Future Charge-Offs

	(1)	(2)	(3)	(4)
*Moderating Variable:*	High Governance Score	Independent Board	Low Inside Ownership	High Gender Diversity
*Manager LLP Style:*	High Discretion	High Discretion	High Discretion	High Discretion
*Dependent Variable:*	CO _t+1_	CO _t+1_	CO _t+1_	CO _t+1_
Manager LLP Style * ALL * Moderator	0.370***	0.235*	0.219**	0.327**
	(0.005)	(0.069)	(0.014)	(0.031)
Manager LLP Style * Moderator	−0.007***	−0.003	−0.003*	−0.005**
	(0.005)	(0.213)	(0.097)	(0.010)
Manager LLP Style * ALL	−0.299***	−0.101	−0.254***	−0.156
	(0.000)	(0.371)	(0.001)	(0.293)
ALL* Moderator	−0.151	−0.063	−0.202***	−0.405***
	(0.208)	(0.416)	(0.010)	(0.000)
Manager LLP Style	0.009***	−0.001	0.008***	−0.001
	(0.000)	(0.444)	(0.000)	(0.462)
Moderator	-	0.001	-	0.006***
		(0.265)		(0.000)
Controls	Yes	Yes	Yes	Yes
N	9,012	7,785	10,515	12,344
Adj. R^2^	0.655	0.733	0.635	0.644
Bank and Year FE	Yes	Yes	Yes	Yes

Table 9 shows regression results for the moderating effect of bank governance and top management team diversity on the influence of individual managers on the predictive value of banks’ loan loss allowances for realized loan charge-offs one year ahead (CO_t+1_). Manager LLP Styles are determined from the manager fixed effects estimated in Table 4 Panel B. We employ a binary variable that takes the value of 1 if the manager fixed effect is above 0 and 0 otherwise (High Discretion). We use five measures for corporate governance as defined in Appendix A and p. 21. High Governance Score is a binary variable that takes the value of 1 if a bank is below the median number of shareholder-unfriendly governance provisions in the year 2006, which is the last year of the IRRC data coverage (Gompers et al. 2003). Independent Board is a binary variable that takes the value of 1 if the bank’s fraction of directors that joined after the CEO took office (tenure-weighted) is equal to or below the median in the year (Coles et al. 2014). Low Insider Ownership is a binary variable that takes the value of 1 if the bank’s average top management team ownership percentage is below the median. High Gender Diversity is a binary variable that takes the value of 1 for top management teams with above or equal to the yearly median gender diversity. We define gender diversity using the Blau index. The index is equal to the sum of the squared proportions of managers in each of the two gender categories. We standardize the Blau index to have a mean of zero. The controls are equivalent to Table 8 (ALL, size, loan growth, and Tier 1) and are defined in Appendix A. P-values are based on robust standard errors clustered by bank. ***, **, and * indicate statistical significance at the 1%, 5%, and 10% levels (two-tailed), respectively

Like other bank policies (e.g., Hagendorff et al. 2010) and studies of the nonfinancial sector (e.g., Karamnou and Vafeas 2005; Qi et al. 2018; Zhang 2019), our results suggest that governance can help moderate the impact of individual management styles on a bank’s reporting policies. To put the magnitude of the moderating effect of the governance variables into perspective, prior research documents that board independence can reduce the sensitivity of manager turnover to performance by nearly two-thirds (Coles et al. 2014)—an effect of only slightly smaller magnitude than the moderating effect observed in our analysis.

## Robustness tests

Appendix B presents additional robustness tests. We perform the AKM analysis with different sample restrictions. Columns 1 and 2 document that our finding of a significant manager fixed effect is unaffected by restricting the sample to the largest connected group and to excluding the largest connected group. Columns 3 and 4 document that the results regarding individual managers are also insensitive to the exclusion of lower-ranked managers. The explanatory power of manager fixed effects remains largely constant (with a partial R2 of 18.9% and 19.1%, p-value < 0.01) if we include only CEOs and CFOs or, more generally, only executive suite-level managers (including CEOs and CFOs) in our sample. The portion of the model R2 explained by the manager fixed effects even increases to more than 59% (compared to 49%) in these samples, underscoring the particular importance of CEOs and CFOs in the decisions about loan loss provisions.

Column 5 presents results for additional tests employing only plausible exogenous turnovers that are less likely to be a result of a bank replacing a manager with a certain loan loss provisioning style.^11^ In this test, we can identify 590 manager fixed effects, and the partial explanatory power of manager fixed effects for discretionary loss provisioning increases to 38% (p-value < 0.1).^12^

In Column 6, we confirm our results by employing a mobility sample that only includes moving managers (mover method), reducing the sample size to 213 identified manager fixed effects. Here, the identification of manager-specific effects rests on the actual movements of managers (e.g., Bamber et al. 2010; Bertrand and Schoar 2003) and, other than under the AKM method, not on their connectedness to moving managers. The results from the mover method confirm the statistical significance of manager fixed effects in loan loss provision models (at the 1% level). However, the fraction of the total variation in loan loss provisions that is explained by manager fixed effects is lower (13.67%) because the mover method assigns greater weights to bank-fixed effects. Consistent with prior literature, the main economic implications of our findings are thus identical, irrespective of whether we use the AKM or the mover method.

To further strengthen the validity of our identification of manager fixed effects and rule out the possibilities that these effects are simply attributable to random associations between individual managers and banks’ accounting patterns, we follow Fee et al. (2013) and Wells (2020) and randomly assign managers to sample banks in each year to arrive at a randomized connectedness sample on which we can re-estimate the manager fixed effects using the AKM method. (See also Bhat et al. 2022.) We repeat this procedure 1,000 times and compare these randomly generated bootstrapped fixed effects to those estimated in Table 4 Panel B. We find that the mean of the bootstrapped manager fixed effects is (jointly) statistically different from our estimated manager fixed effects in Table 4 Panel B (p-value < 0.001), rendering the possibility that our estimates of manager influence over loan loss provisions were just capturing random data patterns much less plausible.

In untabulated robustness tests, we also confirm that our estimation of Eq. (7), which includes state and year fixed effects (see the main result in Table 7 Panel B), is robust to the inclusion of alternative fixed effects used in the literature (i.e., bank type, census region, and year) employing a similar timeliness measure. Our results appear robust to these alternative designs. The same goes for alternative choices of standard errors in Tables 6, 7, and 8 (i.e., nonclustered robust standard errors and robust standard errors clustered by spells of manager-bank pairs).

Another set of untabulated robustness tests examines the potentially varying role of managers’ individual provisioning styles during different periods of our sample (during the 2008–2009 recessionary periods, before and after the enactment of the Dodd-Frank Act, and during the COVID-19 pandemic). Overall, the tests suggest that the main results hold throughout all these periods; that is, managers with a highly discretionary provisioning style are associated with higher absolute discretionary accruals, less timely loan loss provisions, and lower predictability of future loan charge-offs during the entire sample period. However, while we cannot detect any systematically incremental effects before and after the enactment of the Dodd-Frank Act and during the COVID-19 pandemic, the tests reveal an incremental effect during the recession periods when the association between a highly discretionary provisioning style and absolute discretionary accruals increases by a factor of 2, with corresponding and consistent changes for the association between individual provisioning styles and the informativeness of loan loss provisions.

Collectively, these additional tests underscore that the key results of our base model, which uses the AKM method, are insensitive to critical research design choices in our estimation of the role of individual managers in determining banks’ loan loss provisioning.

## Conclusion

This study examines the roles of individual managers in bank loan loss recognition. While prior literature documents that bank-specific incentives and variation over time shape loan loss provisions, we demonstrate a significant influence of particular managers on this major accounting choice. Our tests reveal that these managers exert influence in an idiosyncratic way through their preferences, skills, or values, which are inherently difficult to measure but crucial to understanding the role of managers in the banks’ accounting processes.

Exploiting a large sample of connected managers and banks, we document that, after accounting for bank and time differences, manager characteristics explain approximately 19 percent of the variation in loan loss provisions. This proportion is larger than it is for time-invariant bank characteristics, indicating that, even in heavily regulated sectors such as financial services, managers have the opportunity to shape reporting choices, at least if the tasks are complex and require specific knowledge and the incentives are high. Although there is no direct comparison possible between accounting discretion in financial and nonfinancial industries because of the very different nature of the underlying accruals processes (Stubben 2010), the results tend to indicate that individual bank managers explain more of the variation in discretionary accounting choices than their peers in other industries (approximately 19% compared to 9% in nonbank organizations; Wells 2020). The relation is reversed for firm-fixed effects (approximately 8% in our banking setting compared to 19%; Wells 2020). Because bank managers who have a distinct impact on loan loss provisioning exert their influence on other bank actions in a systematically related way, we use a variety of robustness checks and additional sample limitations to corroborate the main findings and to document that manager-fixed effects are not a mere outcome of bank policy changes.

Our findings also suggest that managers exert their discretion over loan loss provisions opportunistically and less so for informational purposes. In particular, management discretion is associated with a lower timeliness of the provisions and a lower informativeness of the allowance. Given that these properties of loan loss provisions are associated with several outcomes, such as downside tail risk (Bushman and Williams 2015) or lending during crisis periods (Beatty and Liao 2011), the results offer some support for regulatory interventions in banks’ appointments of individual managers. However, we also document that the influence of managers’ individual discretion on banks’ loan loss provisioning policies can be attenuated by corporate governance arrangements. These results imply that supervisors should evaluate management appointments not in isolation but rather within the broader context of the overall incentive structure and composition of a bank’s top management team.

## Data Availability

Data used in this study are obtained from public sources identified in the text.

## APPENDIX A

**Table:** Variable Definitions

Variable	Description	Source and computation
*Manager Characteristics*		
Age	Age in years	ExecuComp: Age of a manager in years
Tenure	Duration of the employment on the current position	ExecuComp: Counts the t-th year that the executive works for the respective bank
Female	Indicator variable for female managers	ExecuComp: Binary variable that takes the value of 1 for female managers
Salary	Fixed salary	ExecuComp: Natural logarithm of fixed salary
Bonus	Bonus compensation	ExecuComp: Natural logarithm of bonus
Total Compensation	Total compensation	ExecuComp: Natural logarithm of total compensation that includes total salary, bonus, other compensation, restricted stock grants and long-term incentive compensation
CEO	Indicator variable for manager’s occupation	ExecuComp/BoardEx: Binary variable that takes the value of 1 if a manager is a CEO in the respective year
CFO	Indicator variable for manager’s occupation	ExecuComp/BoardEx: Binary variable that takes the value of 1 if a manager is a CFO in the respective year
Other C-Level Executive	Indicator variable for manager’s occupation	ExecuComp/BoardEx: Binary variable that takes the value of 1 if a manager is a CRO, CIO or COO in the respective year
*Manager LLP Style:*		
Manager Fixed Effect (continuous)	Continuous manager fixed effect	Self-constructed: Continuous manager fixed effect estimated in Eq. (1), standardized according to Cornelissen (2008)
Highest Discretion Manager	Indicator variable for highest manager fixed effects	Self-constructed: Binary variable that takes the value of 1 if a manager’s standardized fixed effect is in the top quartile of all manager fixed effects estimated in Eq. (1)
High Discretion Manager	Indicator variable for high manager fixed effects	Self-constructed: Binary variable that takes the value of 1 if a manager’s standardized fixed effect is above the median of all manager fixed effects estimated in Eq. (1)
Lowest Discretion Manager	Indicator variable for lowest manager fixed effects	Self-constructed: Binary variable that takes the value of 1 if a manager’s standardized fixed effect is in the bottom quartile of all manager fixed effects estimated in Eq. (1)
*Bank Characteristics*		
Size	Size	Compustat: Natural logarithm of total assets. Measured at the beginning of the period
LLP	Loan loss provisions	Compustat: Loan loss provisions scaled by lagged total loans
Tier 1	Tier 1 regulatory capital	Compustat: Natural logarithm of Tier 1 capital. Measured at the beginning of the period
NPL Ratio	Nonperforming loans	Compustat: Nonperforming loans scaled by lagged total loans
CO	Charge-offs	Compustat: Net charge-offs scaled by lagged total loans
ALL	Allowance for loan losses	Compustat: Loan loss allowance scaled by lagged total loans
EBP	Earnings before loan loss provisions	Compustat: (Earnings + loan loss provisions) scaled by lagged total loans
Loans/Deposits Ratio	Loans-to-deposits	Compustat: Total loans scaled by total deposits
Loan Ratio	Loans to total assets	Compustat: Total loans scaled by total assets
Loan Growth	Change in total loans	Compustat: Change in total loans divided by lagged total loans
Non-Interest Income	Non-interest income	Compustat: Non-interest income divided by the sum of non-interest income and interest and related income
Total Volatility	Total market-based volatility	CRSP: Volatility of realized returns using the market model
ΔCSRET	Case Shiller Return	Federal Housing Finance Agency: Yearly change in the Case-Shiller Return Index
ΔGDP	Gross Domestic Product	Federal Reserve Bank of St. Louis: Yearly change in gross domestic product
ΔUNEMP	Unemployment rate	Federal Reserve Bank of St. Louis: Yearly change in unemployment rate
Timely LLP	Loan Loss Provision Timeliness	Own computation based on Beatty and Liao (2011). We first measure timeliness in our quarterly dataset (Table 1 Panel B), estimating Eq. (5) and (6) using 12-quarter rolling windows, requiring banks to have data on all 12 quarters. We then subtract the R^2^ of Eq. (6) from the R^2^ of Eq. (5) as our measure of timeliness. We classify a bank as timely if the bank is above the quarterly median for more than two quarters in the year
Absolute DLLP	Discretionary Loan Loss Provisions	Own computation based on Beatty and Liao (2014) and Nicoletti (2018). Absolute average residuals from estimating Eq. (2) on the manager-year level
High Governance Score	Governance index	IRRC, Gompers et al. (2003): Binary variable that takes the value of 1 if the bank’s median governance index (G-Index) is below the median G-index in the year 2006, which is the last year of the IRRC coverage
Independent Board	Board Co-option	Coles et al. (2014): Binary variable that takes the value of 1 if the bank’s yearly fraction of directors that assumed office after the CEO (tenure-weighted) is below the yearly median
Low Inside Ownership	Top Management Team Inside Ownership	ExecuComp: Binary variable that takes the value of 1 if the bank’s average top management team ownership percentage during the sample period is below the median
High Gender Diversity	Top Management Team Gender Diversity	ExecuComp: Binary variable that takes the value of 1 for top management teams with above or equal to the yearly-median gender diversity. We define gender diversity using the Blau index that equals${\sum }_{i=1}^{2}{p}_{i}^{2}$, where p is the proportion of firm i’s managers of each gender in the top management team-year. We standardize the measure by subtracting the mean and dividing by the standard deviation

## APPENDIX B

**Table:** Additional Robustness Tests

	(1)	(2)	(3)	(4)	(5)	(6)
	Only the Largest Connected Group	Without the Largest connected group	Only CEOs and CFOs	Only Executive Suite Level	Exogenous Turnover Sample	Mover Method
adj. total R^2^	38.00%	61.00%	32.00%	34.00%	64.96%	63.93%
Partial R^2^ explained by manager FE	16.77%	37.41%	19.10%	18.90%	33.78%	8.74%
% of Model adj. R^2^ explained by manager FE	44.13%	61.32%	59.68%	59.05%	52.00%	13.67%
Partial R^2^ explained by bank FE	8.69%	1.30%	3.65%	4.15%	3.37%	16.76%
% of adj. R^2^ explained by bank FE	22.87%	2.13%	11.42%	12.96%	5.19%	26.22%
Partial R^2^ Residual and Time Fixed Effects	74.54%	61.29%	77.25%	76.96%	62.86%	74.49%
*Statistical Significance of Manager Fixed Effects*						
F-test bank FE = 0	1.60***	0.93	1.47***	1.89***	1.82***	4.54***
F-test manager FE = 0	1.30***	6.23***	1.28***	1.32***	3.09***	2.08***
F-test manager and bank FE = 0	2.45***	1.61***	2.01***	2.00***	5.52***	4.66***
Number of Identified Bank Effects	133	22	68	80	27	155
Number of Identified Manager Effects	1752	447	843	1070	590	213

Appendix B repeats the analysis from Table 4 Panel B employing additional sample restrictions to validate the robustness of the estimated manager fixed effects. The table presents the Shapley decomposition of the variance explained by manager fixed effects, bank fixed effects, year fixed effects, and residuals when estimating Eq. (1). Partial R^2^ explained by manager fixed effects is the percentage of the variation in the dependent variable explained by the manager fixed effect. % of Model adj. R^2^ is the proportion of the model adj. R^2^ explained by the manager fixed effects. Column (1) includes only the largest connected group in the sample, corresponding to group 2 in Table 2. Column (2) excludes the largest connected group from the sample. In Column (3), we estimate manager and firm fixed effects on loan loss provisions in a sample employing only CEOs and CFOs. Column (4) includes all C-level managers in the sample. In Column (5), we use a sample of plausibly exogenous turnovers, including only manager turnovers in the sample occurring after the age of 60. Column (6) estimates manager and firm fixed effects using a sample that only includes managers that switch between banks at least once. Statistical significance of the fixed effects is based on an F-test. The number of banks in Column (1) is 134, with one bank serving as a reference bank in each AKM estimation group (134—1 = 133). ***, **, and * indicate statistical significance at the 1%, 5%, and 10% levels (two-tailed), respectively

## Footnotes

1 For instance, the European Central Bank (ECB) reviews the qualifications, experience, and reputation of newly hired top managers as part of its Fit and Proper Assessment (ECB 2018). Management is also an important assessment category in the CAMELS rating by supervisory authorities in the United States. And the FDIC lists management qualifications and traits as an important assessment category of its own. Concurrent research underscores that these supervisory assessments of the quality of individual managers have important implications for bank performance (e.g., Arif et al. 2023; Cowhey et al. 2022).

2 However, the influence of individual bank managers on loan loss provisioning is relatively small compared to their influence on other bank policies. For example, such managers’ idiosyncratic attributes explain more than 50 percent of the variation in compensation policies (Graham et al. 2012).

3 ExecuComp covers all banks that were included in the S&P 1500 for at least one year, and it contains data available for periods from 1992 onward.

4 Compared to other studies relying on the AKM method, we observe more managerial mobility in our sample; see Sect. 4.2.

5 This procedure first standardizes the mean bank and manager fixed effect within each of the AKM estimation groups to zero by subtracting the average group bank and manager effect. After this normalization, we measure manager and bank fixed effects relative to the group means.

6 Most of the increase in the raw R^2^ is attenuated by the degrees-of-freedom adjustment for the large number of manager fixed effects.

7 In additional untabulated robustness tests, we document that our results are robust to the three other models from Beatty and Liao (2014), indicating that our results are not an artefact of our specific discretionary accrual model.

8 This measure is also used in other recent studies, such as Wheeler (2019), Bhat et al. (2019), and Gallemore (2023).

9 The coverage of the IRRC data ends in 2006 after the merger with Institutional Shareholder Services that led to substantial changes in data definitions.

10 In untabulated robustness tests, we confirm that the results are insensitive to controlling for the number of top managers per bank-year in this analysis, addressing the concern that the number of top managers is positively correlated with the diversity score and negatively correlated with the magnitude of the manager fixed effects, potentially giving rise to a mechanical relationship.

11 We collect data on plausibly exogenous manager retirement turnovers. We consider turnovers that are either classified as “retirement” in ExecuComp or happen at the age of 61 or older as routine turnovers (Custodio and Metzger 2014).

12 In addition, our results on the role of individual managers in determining the overall level of discretion, timeliness and informativeness of loan loss provisions (i.e., Tables 6, 7, and 8) are generally robust to restricting the sample to only CEOs and CFOs; only the results on the informativeness of loan loss provisions about future charge-offs appear less statistically significant while going in the same direction.
